# 725 Case Series: New Porcine Placental ECM for Burn Injuries

**DOI:** 10.1093/jbcr/irac012.279

**Published:** 2022-03-23

**Authors:** David G'sell, Jeffrey E Carter, Nicole M Kopari, William L Hickerson

**Affiliations:** University Medical Center New Orleans, New Orleans, Louisiana; University Medical Center- New Orleans, New Orleans, Louisiana; Children's Hospital New Orleans, New Orleans, Louisiana; Spectral MD, Avita Medical, AccessPro Med, Memphis, Tennessee

## Abstract

**Introduction:**

Human amniotic membrane (HAM) has been used as a biologic dressing for burn wounds since 1955 but limited due to availability, size, and processing costs. In 2021 a new porcine placental product was FDA-approved overcoming challenges with human-sourced products. Our study is the first case series to report outcomes using porcine placental extracellular matrix (PPECM) in the use of adult burn patients.

**Methods:**

Adults with thermal burns resulting in partial-thickness burn wounds (PTBW) were consented and included in the study from 03/2021 to 09/2021. Patients with full-thickness injures, concomitant trauma, or adverse beliefs to porcine products were not included in the study. Serial still images and initial wound measurements were obtained intraoperatively and post-operatively. PPECM trial product processed with a proprietary decellularization method to produce single sheets up to 15x20cm was approved by the facility value assessment committee. Adverse events were defined a priori as infection, increased pain or itching relative to adjacent autografts, or failure to heal. Infection was defined as a PPECM treatment site requiring any change from standard of care or initiation of local or systemic antibiotics. Pain was assessed using a visual analogue scale. Itching was assessed at discharge and follow-up. Healing was assessed using the FDA guidance for wound closure with 2 consecutive visits 2 weeks apart demonstrating 100% epithelialization without drainage or dressing requirements.

**Results:**

Four patients were treated during the study period with wounds involving the torso and major joints such as the hands/wrists and knees. None of the PPECM wounds demonstrated failure to heal or required revision excision, or autograft. None of the PPECM wounds had evidence of infection. PPECM wounds had decreased pain/itching relative to adjacent burn wounds which were treated with split-thickness autograft, autologous skin cell suspension, or allogeneic cultured skin substitute (VAS mean 1 vs 3.1). Healing was noted in all wounds at 1-week primary dressing removal with confirmation at 2-week interval follow-up.

**Conclusions:**

PPECM treatment of PTBW was not associated with adverse events and resulted in favorable outcomes clinically. The large size, ease of use, and lower costs relative to HAM is an intriguing alternative for PTBW. Comparative studies are needed in the field to determine best practices and overall value.

